# A method for complete plant taxon and site inventories in large forest areas with the help of orienteering maps, as exemplified by target forests in Switzerland

**DOI:** 10.1371/journal.pone.0225927

**Published:** 2019-12-10

**Authors:** André Strauss

**Affiliations:** Mabritec AG, Riehen, Switzerland; University of Waikato, NEW ZEALAND

## Abstract

Complete plant inventories of large areas of forests in the moderate and boreal zone have thus far been infeasible and have also not been published. The use of orienteering maps (O-maps) for sampling for inventories was tested. In the sampling method presented herein, the “O-map/way method”, O-maps were used for controlled and systematic inspection and sampling, making it possible to carry out successfully complete plant taxon and site inventories of large forest areas (1 to 100 ha). O-maps are much more suitable than the best national or similar topographic maps (NT-maps) for plant inventories in forests; O-maps have many advantages (smaller scale/better resolution, better legibility, internationally standardization, information on vegetation and accessibility), and they contain more small objects, ways (= tracks of any size; roads), and lines and thus have much smaller subareas that allow good orientation and systematic screening for plants. For the example of plant taxon inventories in 6 target areas of 25–85 ha of Swiss midland forests in the moderate/colline zone, the O-map/way method (all accessible areas are screened) was shown to be clearly superior to alternative sampling methods (partial areas screened), such as the NT-map/way method or a plot method, in which only 79.6±6.7% or 34.5±6.6%, respectively, of the taxa found by the O-map/way method were recorded. Taxa detected only by the O-map/way method were shown to be relatively rare at a local as well as at a national scale. The O-map/way method could also be successfully applied to the inventory of plant sites in large forest areas: As shown by the distribution of the sites of five plant species in a target area of 30.1 ha, the great majority of plant sites were detected only by the O-map/way method; but only a few sites were detected by the alternative methods. As O-maps for forests are widely available in many countries, the O-map/way method might allow for complete inventories and other studies in large forest areas.

## Introduction

Inventories of plant taxa have been the subject of studies for many years, especially in cities and their surrounding regions [1], and serve as the basis for numerous applications, such as local biodiversity assessments, landscape planning, or studies of the dynamics of plant distribution. Methods for inspection and sampling to establish plant inventories in urban areas can be rather easily performed by searching for plant taxa along streets and in the relatively small areas with vegetation in the settlement zones. However, a complete (herein meaning that every accessible place of the target area is screened) and careful establishing of inventories of plant taxa or plant sites in forest areas of 1–100 ha is nearly infeasible with the existing methods because of increasing difficulties in orientation for inspection for the investigator or an enormous workload and has not, to our knowledge, been published so far for target forests, at least in the moderate and boreal zones. However numerous reports exist on plant studies of forest areas < 1 ha using different versions of the plot sampling method (herein called the “Plot method”) [2,3]. Plant inventories in forest areas > 1 ha along ways (= herein tracks of any size; roads) are occasionally done [4], but are not complete, because only a portion of the target area is screened. This lack of “complete” plant inventory studies for larger contiguous forest areas is at least partly due to the insufficient resolution of the best conventional topographic maps available for inspection of forest target areas. Even one of the most suitable national or similar topographic maps that covers forest, rural and urban parts (from now on called “NT-maps”), used herein for comparison, the “Schweizer Landeskarte” from swisstopo, at a scale of 1:25 000 [5], would not allow a sufficient orientation for the investigators for complete inspection in larger forest areas to perform plant inventories with a reasonable amount of efforts.

We suppose that the use of orienteering maps (from now on called “O-maps”), produced for sport competitions in “orienteering” [6,7,8,9], would be much more suitable for plant inventory studies in forests than NT-maps, as they are superior in many aspects (scale usually 1:10 000, more legible, give information on vegetation, contain more details such as ways and small objects, and consequently have thus smaller areas between ways and would allow investigators to navigate more easily). Furthermore, most of the O-maps have the same display and show the same high resolution worldwide according to the rules of the “International Orienteering Federation” [7]; in contrast to NT-maps, O-maps would thus allow a better global comparison for plant inventory studies in forests. O-maps are generally updated every 5–10 years. New O-maps are geo-referenced and are thus compatible for comparison and combinations with other maps. The distribution of O-maps is considerably wide, especially in central and northern Europe, where 500–2 500 different O-maps of forests exist per country exist (e.g. Germany, in 18.03.2019 had 2 313 O-maps; [10]). In 2016, Switzerland had approximately 870 listed O-maps for different forests [11], with a total area of forest of approximately 5 100 km^2^, corresponding to approximately 40% of the total forest area of Switzerland (calculated from data of BAFU [12]). Even outside Europe, many O-maps (not all with forest and some repeatedly) exist as shown by WorldofO.com on 12.8.2019 [9], e.g. for the USA 3 170, Canada 544, Australia 3 427, and New Zealand 1 892, and even for China 86, Japan 38, Morocco 33 and South Africa 34. If no O-map is available for a forest target area, mapping companies, that exist in many countries of the world (e.g. the USA: https://www.orienteeringusa.org/mappers), could produce an O-map or a map of a similar standard for a plant inventory study. New O-maps are available in paper and electronic forms from the producing orienteering clubs, whose addresses can be found on the internet or can be obtained from the corresponding national orienteering federation. The use of electronic forms of O-maps is dependent on map drawing programs (e.g. OCAD; www.ocad.com). Examples of O-maps can be found on the internet [8,9].

The aim of this study is to test the usefulness of O-maps for plant inventory studies in forests (which are the basis for local biodiversity studies) at the example of six forest target areas of a size of 0.23–0.90 ha in the midlands of Switzerland, near Berne. The usefulness of O-maps and conventional NT-maps will be compared with respect to map features and applicability to plant taxon inventory and plant site inventory studies. As an outcome, a sampling method (“sampling” includes here also inspection) for successfully performing plant inventory studies for large forest areas using O-maps, the “O-map/way method”, will be presented and compared to other methods.

## Materials and methods

### Topographical maps

The paper topographical maps used for this study, which contained the 6 target areas were 4 O-maps), provided with permission from OLG Bern [13] and the most suitable national topographic NT-map from swisstopo [5] as specified in S1 Table. Specifications of the map features and symbols on the O-maps were given in ISOM 2017 by International Orienteering Federation [7]. Electronic forms of the used O-maps were obtained on request from www.olgbern.ch and applied for the analysis of data using the program OCAD 12. An electronic access of the used NT-map, however in an updated edition, can be found at https://map.geo.admin.ch/. For inspection and sampling, all the maps were enlarged on paper to a scale of 1:5 000.

### Target areas

The 6 target areas used here are individually contiguous parts of forest in square kilometres for an ongoing inventory study of the flora of the City of Berne, FLIB. As given in S1 Table, they have area sizes for the inventory areas (CA2corr) of 30.19–88.42 ha (hectares) and are located in 4 different forests; one forest (“Könizberg”) contains 3 adjacent target areas (809f, 710f, 810f). The 4 forests lie at most 11 km apart; three of them (“Bremgartenwald”, 410f; “Dählhölzli”, 714f; “Könizberg”, 809f, 710f, 810f) lie mainly in the urban region of Berne close to traffic routes and have partly strong anthropogenic influences; whereas the forest “Forst”, 703f lies in a rural region, west of Berne. These forests are otherwise typical Swiss midland forests of the mixed forest type in the moderate/colline zone [14].

**Fig 1:**
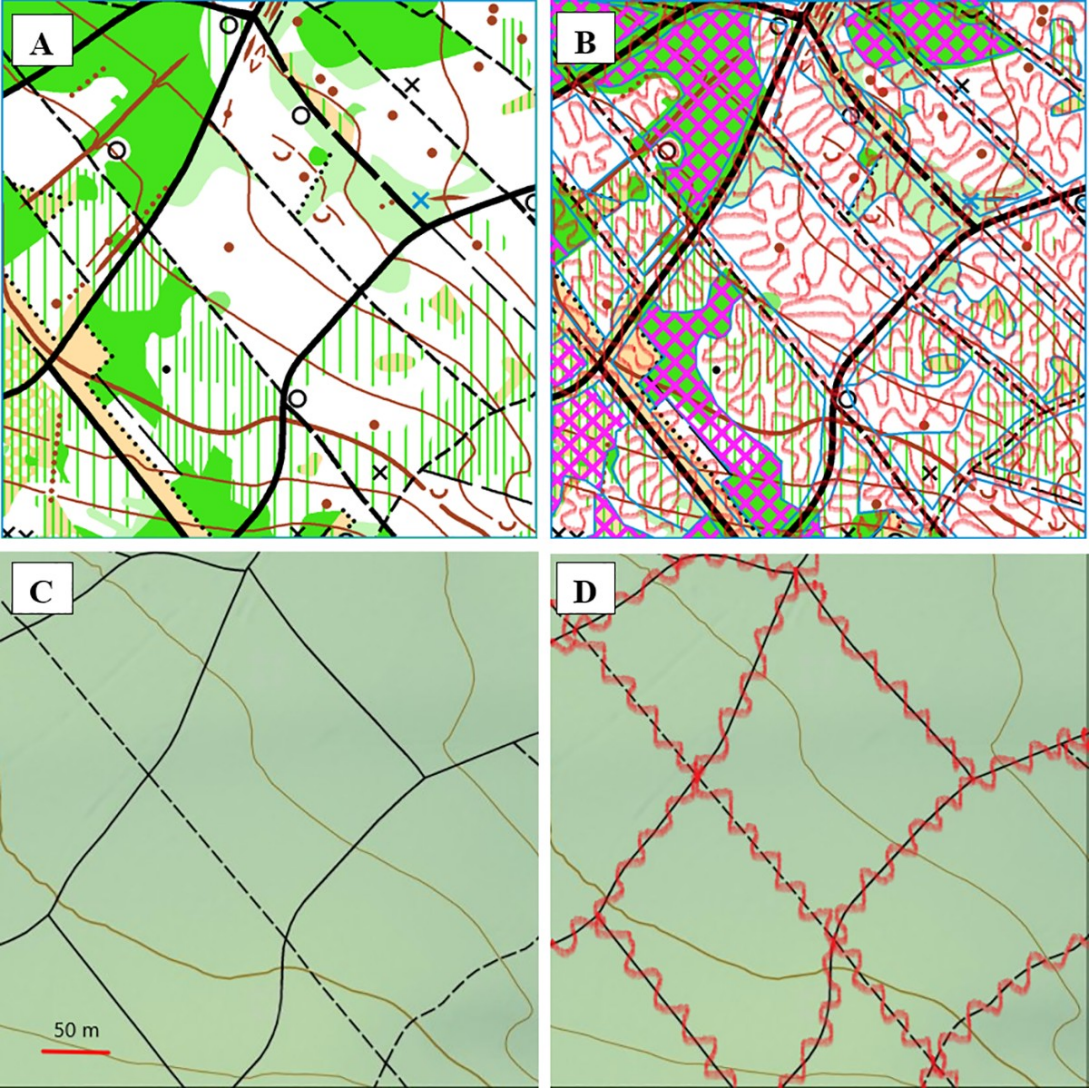
Typical inspection patterns of screening for plant taxa in large forest areas using the O-map/way method or the NT-map/way method. The part shown is a 16.8 ha large area of the target area 710f from the forest “Könizbergwald” (S1 Table). A and B show the area on the O-map (Reprinted from O-map “Könizberg”, #299 Q, under a CC BY license, with permission from OLG Bern, original copyright 2013) and C and D show the area on the NT-map (Reprinted from Landeskarte der Schweiz, #1166, 2012, no license needed, with permission for reproduction from swisstopo, 2019). A and C show the corresponding original map parts; B and D show in a schematic way the meandering inspection routes (in red), in B along and between all ways, and in D along only NT-ways. In B, reduced accessible areas are shown as crossed lines in magenta. Features are explained for O-maps by IOF, [7], for NT-maps, by swisstopo, [5].

Over the course of the inspections, the target areas (TA) were split into two compartment areas as follows: CA1: not accessible for inventory (fenced in, buildings, railway tracks, rocks, etc.) or free from vegetation (e.g. asphalt roads or places); and CA2 (= TA–CA1): forest with natural ground, accessible for inventory. CA2 was further subdivided into the two sub-compartments CA2a (fully accessible by foot) and CA2b (limited access because of very dense vegetation; partly visible) (see also Fig 1). Because the lack of light and the dense presence of competing plants make the discovery of other taxa very unlikely, CA2b was included in the accessible inventory area. In this study, the inventory areas were slightly reduced because a 2 m broad forest edge hem was subtracted to improve of comparability between target areas, giving the corrected inventory area CA2corr. The area sizes for the target areas (TA) and the compartments and sub-compartments are given in S2 Table.

### Statistical evaluation of the data

In general, the variability of numbers and frequencies was assessed by using the arithmetic mean (mean) and empirical standard deviation (SD). However, a correct statistical comparison of the numbers and frequencies of taxa per area was not possible because of missing proportionality between the taxon number and area, which resulted in saturation curves when the taxa per area were plotted [15]. In this case, the variability of the percentages of taxa for other sampling methods compared to the taxa for the O-map/way method was assessed in approximation also as mean and SD.

### Site localization, area size and way length

The geographic locations of the plant sites for plant taxon and plant site inventories was determined by using a Garmin Dakota 20 GPS instrument and by drawing on the O-map. The accuracy and variability of the GPS data were previously assessed by performing 126 measurements in the forest in the target area 714f at 7 locations, under different conditions. The mean accuracy of the GPS instrument was 6.9 m (n = 42); the variability was 4.9 m (n = 126). Localization by drawing on the O-map was used for comparison and for avoiding errors; features on the O-maps close to the sites served for a sufficiently good localization of sites (see below). The coordinates (Swiss Grid) were determined in metres. The area sizes and way lengths were measured on O-maps twice electronically using the program OCAD 12. The O-maps contain all the ways present on the NT-map, but additionally those, which are only present on the O-maps.

### Plant taxon identification, inspection and sampling

Plant taxa were identified according to the 5^th^ edition of Flora Helvetica [14], where 3 117 plant taxa (Pteridophyta, Gymnospermae and Angiospermae) that are present in Switzerland are described. Sampling was also performed for plant taxa in the same range of taxonomy. In 70% of the observed taxa, the identification was clear and was therefore done on the site; in the remaining 30% of less clear cases, the plant material was collected and herbarized and/or photographed and identified later, using the appropriate means. A few rare cases, in which an identification was not possible, were not included in the evaluation. The inspection and sampling were carried out by the same person in three seasonal inspection rounds (March-April; May-June; August-October) in 2014 and 2015, partly in course of an ongoing inventory study of the flora of the City of Berne, FLIB, applying the O-map/way and NT-map/way methods. The inspection and sampling applying the Plot method was performed in 2016 in a similar way. Except for the Plot method, plant taxa were recorded for this study separately for all three inspection compartments (along NT-ways = ways on the NT-map; along O-ways = ways only on the O-maps; between all ways). For each new taxon in each of the three inspection compartments, the site location was determined by GPS and sketched in the O-maps and the abundances were recorded in classes of 1–25, 26–1000 and > 1000 plants for the inspection area. The time requirements for the inspection and sampling of the three sampling methods used herein were recorded separately and expressed in hours/km^2^ for the three seasonal inspections (S3 Table). Inspection times were related here to CA2acorr, because time was spent only in this fully accessible sub-compartment (S2 Table).

### O-map/way method for taxon inventory

The “O-map/way method” described herein is defined as a sampling method for plant taxon inventories in forests, using O-maps for inspection and screening the total, accessible target forest area in a complete way, meaning on all accessible places along and between all ways (Fig 1). The method is also documented under http://dx.doi.org/10.17504/protocols.io.6xbhfin. The general process for searching for taxa with the O-map/way method in the given target area is as follows: three seasonal rounds of inspection/sampling are conducted in the same year by navigating with the help of the 1:5 000-scale paper O-map and with compass and GPS in a first step along larger ways (present on the NT-map), then in a second step along smaller ways (present only on the O-map) and for completion finally in a third step by meandering in 5–20 m broad loops between all ways and other recognizable lines on the O-map (paths, tracks, fences, vegetation boundaries, water courses, earth walls, gullies, etc.) so that every accessible place can be searched for new taxa (as shown in Fig 1).

For this study, the criteria for recording taxa along ways, were to record all new taxa for this study on both sides of the way for herbaceous and small plants within 3 m of the way, for at least 1 m high shrubs within 10 m of the way, for at least 2 m high trees or clearly visible flowering plants within 20 m from the way; for all three compartments (along NT-ways, along O-ways and between ways) new taxa were recorded for this study separately.

For the situation “At NT- and O-ways only”, where taxa were recorded only along NT- and O-ways, only the first two screening steps given above were carried out.

### NT-map/way method for taxon inventory

The “NT-map method” described herein is another defined sampling method for plant inventory in forests, in which only NT-maps are used for inspection and screening only along all ways on NT-maps in the target forest areas. A similar and defined sampling method, called “transect route”, was also used for plant inventory studies in the forests [4]. For the NT-map/way method herein, the inspection and sampling procedure was conducted as given above for the first step of the O-map/way method; however orientation was done with the help of NT-maps, and inspection was only along all NT-ways and the second and third steps were omitted.

### Plot method for taxon inventory

The various versions of the Plot method for inventory studies were reviewed [3]. Here the following procedure was used for the inventory of the plots: For each of the four tested target areas 703f, 410f, 710f and 714f, two rectangular plots with area sizes of 0.5 ha were chosen according to the following criteria: at least 400 m apart from each other; representative for the target area; containing ways; fully accessible by foot. The orientation, inspection and sampling were performed as above given for the O-map/way method by inspecting by foot every part of the plot for new taxa.

### Plant site inventory in forests by O-map/way method

The usefulness of the O-map/way method for studying also the abundance and distribution of sites of given plants in forest areas > 1 ha was tested, using an adapted protocol. For this, the sites of five plant taxa (*Prunus laurocerasus*; *Sorbus aucuparia*; *Pinus sylvestris*; *Athyrium filix-femina*; *Dryopteris filix-mas*) in the 30.1 ha large sub-compartment of the target area 714f (CA2corr, but without a small separated subarea) were simultaneously recorded during two subsequent inspection rounds A and B by the same person in May-June 2017 and August-September 2017, respectively. The 5 plant species were chosen for this study, because on one hand their frequencies in the target areas (sample size of 150–250 sites) and their more or less even dispersion are ideal for this study, on the other hand they represent examples of plants that differ in some aspects (2 herbs, 2 shrubs, 1 tree; differently recognizable on inspection but clear for their identification). The minimum size for recording plants was > 10 leaves for *Prunus laurocerasus*, > 0.5 m in height for *Sorbus aucuparia* and *Pinus sylvestris* and > 0.1 m in diameter for *Athyrium filix-femina* and *Dryopteris filix-mas*. The plant sites included all individual plants of the given taxon within a radius of 5 m. The criteria for recording and classifying plant sites with respect to distance from the ways were the same as given above for the inventory of taxon. The estimated locations of the sites were determined as the centres of the two determined sites in the inspection rounds A and B by GPS as well as by sketching on the O-map. The overall variabilities for the distances of the two determined sites to the corresponding centres of the two sites (n = 88) were 5.3±2.9 m for the GPS records and 2.1±1.4 m for sketching on the O-map records.

The optimized and general protocol for searching for plant sites, which is also given at (http://dx.doi.org/10.17504/protocols.io.6xhhfj6), is similar to the O-map/way method described above for plant taxa. It preferably involves three consecutive rounds of inspection/sampling by different persons with the help of the O-map at a scale of 1: 5 000 on paper (at the time when the given plant can be best recognized) by first recording the presence and locations of plant sites along the NT-ways (present on the NT-map) and along the O-ways (present only on the O-map) and then by meandering between all ways and lines and searching for sites in all accessible places. Site locations are preferably determined by high resolution GPS and sketched in the O-map.

## Results

### Comparison of O-maps to NT-maps with respect to usefulness for plant inventory studies

A comparison was carried out on the sizes and frequencies of the elements on the O-maps and the NT-map that are relevant for performing inventory studies in the target areas in forests. With respect to the subarea sizes, the mean size of the subareas between the lines on the O-maps (0.19 ha) for the tested target areas was 4.5 x smaller than the mean size of the subareas between the ways on the O-maps (0.85 ha) and even 9.5 x smaller than the mean size of the subareas between the ways on the NT-map (1.80 ha). The smaller sizes of the subareas on the O-maps compared to those on the NT-map was manifested for all 6 target areas to a similar degree (Table A in S4 Table). Furthermore, the number and mainly the frequency of the subareas > 1 ha (difficult for inspection) for all 6 target areas were distinctly smaller on the O-maps compared to those on the NT-map (Table A in S4 Table; Fig 1). The O-maps for the target areas also contain with 19.84±7.93 km/km^2^ a 1.7 x denser network of ways/paths than the NT-map, which has a way/path density of 11.57±2.63 km/km^2^ (Table B in S4 Table; Fig 1). Furthermore, the given O-maps contain a much higher amount (mean 206.9±131.4 features/km^2^) of small orientation features compared to the 0 or 1 features/km^2^ for the NT-map (Table C in S4 Table; Fig 1). In addition, the given O-maps comprise better qualitative properties with respect to inspection compared to the NT-map, such as better legibility (forest in white; low scale), density of vegetation shown, and more detailed terrain structures.

### The O-map/way method for plant taxon inventory in forests

Thanks to the above-shown advantages of the O-maps compared to the NT-map, it was possible to establish a procedure of systematic inspection (along NT-ways–along O-ways–between ways/lines in small subareas) for a taxon inventory over the complete, accessible areas for the 6 six forest target areas of with sizes from 30.19–88.42 ha. The resulting “O-map/way method” for complete plant taxon inventories in large forest areas, which is described in detail under “Materials and Methods”, is mainly based on the use of O-maps for inspections. At the same time, it could therefore be successfully applied for the six target areas to the ongoing inventory study of the flora of the City of Berne, FLIB. For all 6 target areas combined, 560 different taxa were found; thereof 103 taxa were observed in all 6 target areas.

The O-map/way method described here was compared to three other sampling methods (“NT-map/way method”, “At NT- and O-ways only”, and “2x0.5 ha-Plot method”) for the observed number of plant taxa at three seasonal inspections in one year. In Table 1 (and the associated raw data thereof in S7 Table) the frequencies of the observed taxa obtained through the four sampling methods in the six target areas are shown in absolute numbers and as percentages of the numbers of taxa found by the O-map/way method. In total, with the O-map/way method, 194–375 (median: 296.8) taxa were observed for the six target areas with area sizes of 30.2–88.4 ha (mean: 68.6 ha). For the “At NT- and O-ways only” method, only 87.0±4.0% of taxa detected by the O-map/way method were found. Due to the consecutive inspection procedure, all these taxa were also observed by the O-map/way method; but on average 13.0% taxa were additionally observed by the O-map/way method compared to “At NT- and O-ways only”. With the NT-map/way method, where only taxa along the NT-ways were recorded, only 79.6±6.7% of taxa compared to the O-map/way method were found. All these taxa were also found with the O-map/way method and “At NT- and O-ways only” method; on average 20.4% of taxa were additionally observed by the O-map/way method compared to the NT-map/way method. With the 2x0.5 ha-Plot method, where the taxa in two representative plots of 0.5 ha size in each of four target areas were recorded, only 34.5±6.6% of the taxa compared to the number from the O-map/way method were found.

**Table 1:** The numbers of plant taxa found during 3 seasonal inspection rounds by different sampling methods.

Sampling method	Numbers of taxa found (in % of taxa by O-map/way method)						
Target area	703f	809f	410f	710f	810f	714f	Mean±SD
Area [ha]	71.5	70.3	88.4	84.4	67.0	30.2	
**O-map/way method**	375 (100)	263 (100)	341 (100)	326 (100)	277 (100)	193 (100)	(100)
**NT-map/way method**	285 (76.0)	222 (84.4)	300 (88.0)	263 (80.7)	221 (79.8)	133 (68.9)	(79.6±6.7)
**At NT- and O-ways only**	320 (85.3)	237 (90.1)	313 (91.8)	286 (87.7)	240 (86.6)	155 (80.3)	(87.0±4.0)
Plot area [ha]	2x0.5		2x0.5	2x0.5		2x0.5	
**2x0.5 ha- Plot method**	112 (29.9)		100 (29.3)	142 (43.6)		68 (35.2)	(34.5±6.6)

Six target forest areas of sizes of 30.2–88.4 ha were tested; the area sizes are the “Accessible corrected” areas CA2corr (S2 Table). The O-map/way method: along and between all ways (complete); The NT-map/way method: only along NT-ways; 2x0.5 ha-Plot method: 2 representative 0.5 ha-plots per target area. The target areas and sampling methods are further specified in the Materials and Methods.

The plant taxa found only by the O-map/way method (only between all ways) were characterized with respect to “relative rareness” on national and local levels: A considerable proportion of these were classified as “relatively rare”, namely 33.8±8.2% were present in less than 40% of the total of 593 subareas that completely cover the area of Switzerland [14], and 74.9±7.4% were present in the abundance class “1–25 individual plants” for the tested target areas (S5 Table, S8 Table).

To estimate the time requirement for inspection and sampling with the taxon inventory applied sampling methods over 3 seasonal inspections in each of the 6 target areas, the times per area (CA2acorr = fully accessible area, corrected) were determined. As shown in S3 Table, the mean time requirement for the O-map/way method (total area) was 244.6±21.1 hours/km^2^, for the NT-map/way method (partial area), 89.4±19.2 hours/km^2^, and for “At NT- and O-ways only” (partial area), 131.9±15.2 hours/km^2^.

### The O-map/way method for plant site inventory in forests

**Fig 2:**
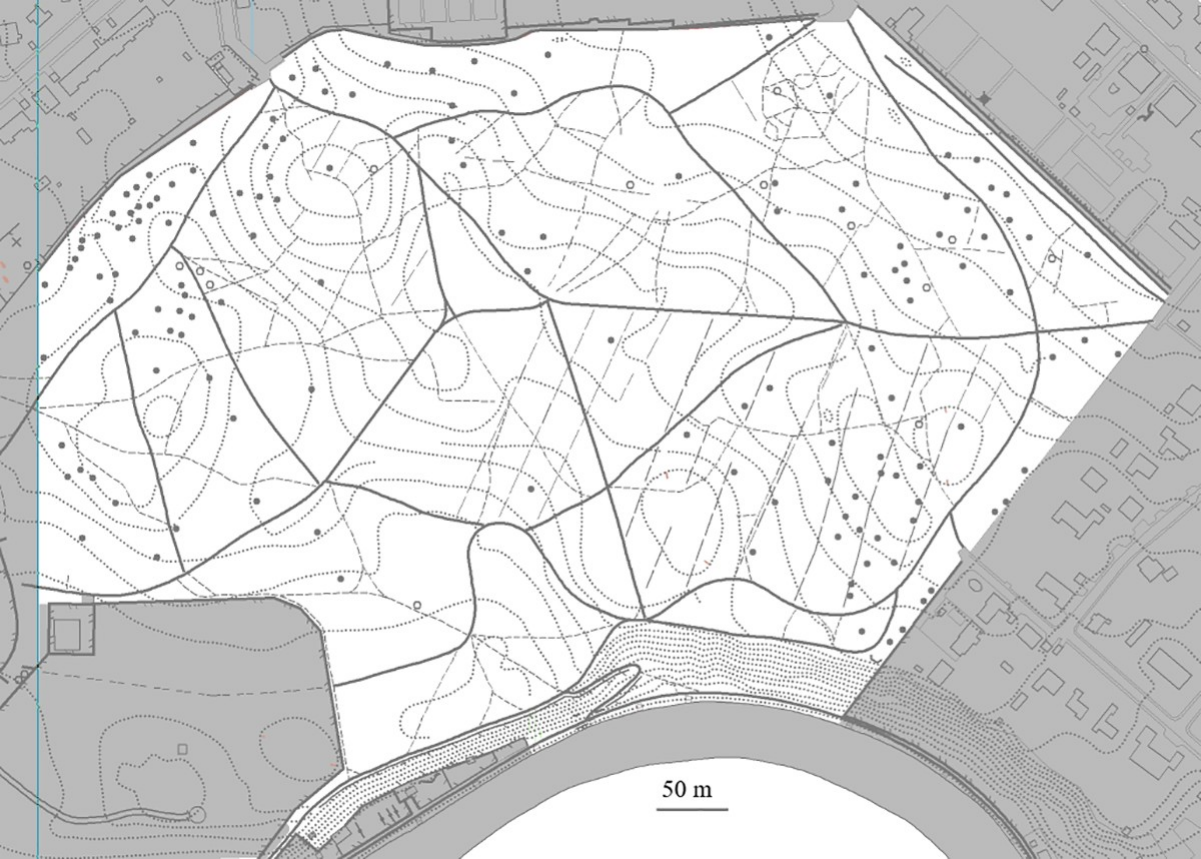
Distribution of sites of *Prunus laurocerasus* recorded during 2 inspection rounds by the O-map/way method (between all ways) or by the NT-map/way method (only along NT-ways). The shown forest area (in white) is a 30.1 ha large part of target area 714f of forest “Dählhölzli” (S1 Table) and is displayed here in a modified and colourless form from the O-map (Reprinted from O-map “Dählhölzli”, #1772 Q, under a CC BY license, with permission from OLG Bern, original copyright 2014). From a total of 152 recorded plant sites, 139 were only recorded by the O-map/way method between ways (black dots). The remaining 13 sites (shown as circles) were also found along the ways. (Table 2). The NT-ways are shown as continuous lines, the O-ways as dashed lines, the forest tracks as lines with long dashes, and the contour lines as dotted lines.

To test if the procedure of the O-map/way method can also be applied to plant site inventories in the larger forest areas, the coherent part and completely accessible area of target area 714f (30.2 ha) was screened in a complete way and simultaneously for sites of the plant species *Prunus laurocerasus*, *Sorbus aucuparia*, *Pinus sylvestris*, *Athyrium filix-femina* and *Dryopteris filix-mas* on two consecutive inspection rounds. An unambiguous identification and localization of all the sites was possible from the records of the two inspection rounds by GPS and sketched on the O-map. By checking the reproducibility of the discovery of sites during two inspection rounds, it was shown that 94.0% of the total of the 199 sites with the well recognizable tree *Pinus sylvestris*, but only 61.2–72.8% of the total of the 98–254 sites of the less recognizable species *Prunus laurocerasus*, *Sorbus aucuparia*, *Athyrium filix-femina and Dryopteris filix-mas* were found on both inspection rounds (S6 Table). The distribution of sites in this target area is shown by the example of for *Prunus laurocerasus* in Fig 2. From the location of the total of sites with respect to the distance to the ways on the O-map and the NT-map, it is evident that the great majority of the sites for all five species are detected only on inspection by the O-map/way method; only 2.0–29.1% of all sites were close to NT-ways and would be detected by the NT-map/way method (Table 2). The time requirement per area (calculated per 1 km^2^) for inspection and sampling by simultaneously recording the sites of the 5 species was 297.9 hours/km^2^ for inspection round A in early summer and 399.7 hours/km^2^ for inspection round B in late summer.

**Table 2:** Number of plant sites found for 5 plant species by different sampling methods.

Sampling method	Number of sites found (in % of sites by O-map/way method)				
Species	*Prunus laurocerasus*	*Sorbus aucuparia*	*Pinus sylvestris*	*Athyrium filix-mas*	*Dryopteris filix-femina*
O-map/way method	152(100)	254(100)	199(100)	98(100)	151(100)
NT-map/way method	3(02.0)	22(08.7)	58(29.1)	9(09.2)	21(13.9)
At NT- and O-ways only	13(08.6)	72(28.3)	109(54.8)	32(32.7)	46(30.5)

Plant site recording was performed at 2 consecutive inspection rounds with simultaneous recording of the sites of the given 5 plant species in a forest target area 714f (30.1 ha) by the O-map/way method (along and between all ways: complete), the NT-map/way method (only along NT-ways) and “At NT- and O-ways only”. The sampling methods are further specified in the Materials and Methods.

## Discussion

A new method is presented here that allows, for the first time, complete plant taxon and plant site inventories of large target forest areas with a reasonable investment of time and workload using orienteering maps (O-maps) for the inspections. The absence of published studies on the complete plant inventories of large forest areas is mainly due to the lack of topographic maps that are sufficient for plant inventory inspections in forests. Compared with NT-maps, O-maps are much more suitable for this purpose, as they have obvious qualitative advantages (smaller scale/better resolution, white background/better legibility, information about vegetation and accessibility). As shown here in the example of typical Swiss midland forests in the moderate/colline zone, O-maps also contain many more small objects, and more ways and thus have consecutively distinctly smaller subareas between the ways and even more accentuated between the recognizable lines than the compared, high-quality NT-maps (S4 Table). In particular, the presence of only a few, slightly larger subareas than 1 ha on O-maps is crucial for their good suitability for the inspection and complete systematic screening for new plant taxa or plant sites for inventories. Complete plant taxon inventories in forest plots up to a target area size of 1 ha are feasible using the Plot method and have often been published [3]. However, with increasing area size—because of obvious difficulties in orientation and systematic screening–complete sampling is becoming increasingly difficult, using the available means, in a reasonable length of time. Also, the use of GPS and/or marking bands, etc., in combination with NT-maps are not feasible for this purpose because of the excessive workload. The use of O-maps is thus far the only feasible method for complete plant inventories in larger target areas of forest, at least in the moderate/colline zone. Furthermore, O-maps have the same high standard, appearance and availability worldwide.

An application of this described O-map/way method for a complete taxon inventory of the 6 forest target areas was found to be absolutely feasible for these types of forests. Compared with the three tested sampling methods used here, the O-map/way method found on average 20.4% more taxa than with the NT-map/way method (only along NT-ways), and 13.0% more taxa than with the “At NT- and O-ways only” method and 65.5% more taxa than with the 2x0.5 ha-Plot method (in 2 plots of 0.5 ha). This shows the clear superiority of the O-map/way method to the other tested methods with respect to the most relevant aspect for inventories, the found taxa numbers and frequencies for the target areas. This is no surprise, as the O-map/way method is the only complete method and contains, in addition to the taxa of the sub-compartments of the compared methods “along the ways”, also the taxa of the sub-compartment “between the ways”.

Although the completeness of taxa achieved in the inventories that used the O-map/way method is not absolute for various reasons (annual and seasonal differences, anthropogenic influences, overlooking, dependence on investigators, etc.), the “degree of completeness” might be similar to that for inventories in urban areas, which have similar limitations. Repeat surveys (partly the case here because of three rounds of seasonal inspections) and different investigators should improve the “degree of completeness”. The registered mean inspection and sampling times at three seasonal different times for the taxon inventory with the O-map/way method was 244.6±21.1 hours/km^2^ for the accessible area and is thus distinctly higher than those registered for “At NT- and O-ways only” and the NT-map/way method, both with much smaller areas. Because of the lack of corresponding data on time requirements in the literature, a comparison to other published values is currently not possible.

Forest accessibility for taxon inventories is another factor revealed and assessed here by the O-map/way method; on average, 24.8±6.0% of the 6 studied target areas were not accessible or had reduced accessibility for inspections. To relate data on taxon numbers and frequencies to the relevant area, these are calculated here for the accessible area, including “reduced accessibility” areas. As mentioned in “Materials and Methods”, there is no proportionality between number of taxa and the size of the area [15], and a statistical comparison of the taxon numbers and frequencies of large forest areas is only approximate, also because compared large target areas of forests are usually of different size and/or have different portions of “not accessible” compartments. A statistically correct comparison of the values of taxon numbers and frequencies of the different target forest areas, also in relation to other factors, can currently be done only for constant target areas, as is the case for a standardized Plot method, but mostly not for the O-map/way method.

Reports on complete plant site inventories for smaller forest areas exist [3], but again to our knowledge none has been published so far for forest areas > 1 ha, or detection of sites was only exceptionally possible for given taxa by specific methods, such as remote sensing. As shown here at the example of the site distribution of the five plant species in a large defined 30.2 ha part of the target area 314f, the adaption of the O-map/way method described herein to the complete plant site inventories for this type of forest, was also shown to be absolutely feasible. When compared to those detected by the other tested methods, it turned out, that the large majority of the plant sites for the 5 plant species were detected only by the O-map/way method, when screening was done for the plant sites in all compartments, along and between ways present on O-maps and NT-maps. Many fewer sites were discovered by the tested alternative sampling methods (Table 2, Fig 2). A statistical evaluation for the site numbers of a given area compared to other target areas or in relation to other factors of interest is in many cases possible, as proportionality did exist between site numbers and area size. The frequencies of repeated detection of the sites in two rounds of inspection were rather low in our study; only sites of the easily recognizable tree *Pinus sylvestris*, were repeatedly detected to a high degree (94.0%), whereas the other four tested plant species, which were difficult to recognize under the used conditions, were recorded in both rounds of inspections by the O-map/way method in only in 60–80% of all found sites (S6 Table). To improve the completeness of the plant site inventory, it is thus especially important for difficult-to-recognizable plants to perform three or more rounds of inspection.

Plant taxon inventories in parts of large forest areas have taken place thus far without completely screening the target forest areas, because this was almost not feasible. The O-map/way method herein presented offers the possibility of performing the inventories in a complete way and thus to increase the scientific quality of these studies. The application of the O-map/way method for plant inventory studies in larger forest areas is demonstrated here at the example of 6 target areas in typical Swiss midland forests in the moderate/colline zone. Five of the target forest areas are in an urban region and contain relatively many ways, facilitating inspections for inventories. However, also for the target area 703f, which lies in a rural region and contains relatively few ways (Table B in S4 Table), the O-map/way method is applicable in the same way. The application of the O-map/way method should thus be possible for the majority of all Swiss midland forests and for many forests of a similar types in other countries at least in the moderate/colline zones, supposing the existence of O-maps, which is often the case. O-maps could, of course, also be helpful in forest areas < 1 ha, e.g. for facilitating the planning and inspection of the plots. Even an application of the O-map/way method to large target areas of open woodland, to mountain forests or forests in the boreal zone, where O-maps are available, might be possible, as other types of lines (small water ways; swamps; detailed terrain structures) on the O-maps could replace ways for allowing controlled systematic inspections for inventories of larger areas.

Application of the O-map/way method on plant inventories in large forests could also contribute to studies on specific aspects of biodiversity, as shown in the example of the high numbers of relatively rare taxa among all taxa exclusively found by the O-map/way method as shown in the present study (S5 Table). The O-map/way method might thus be used to detect relatively rare plant species in forests.

Further technical improvements of the O-map/way method are possible, e.g. by using tablets or smart phones, which are suitable for inspections, with electronic O-maps, plant registration programs, and integrated GPS loaded.

Thus this article describes a method that uses O-maps for inspection/sampling, of complete plant taxon and plant site inventories in contiguous forest areas > 1 ha in the moderate/colline zone of central Europe, and has potential applicability to forests in other regions.

## Supporting information

S1 Table Specification of the target forest areas with respect to location, size and used maps. O-maps edited by OLG Bern: www.olgbern.ch; NT-map edited by swisstopo: www.swisstopo.admin.ch.
(DOCX)

S2 Table Compartmentation of the target areas with respect to accessibility on inspection. Further specification of the compartments is given under “Material and methods”.
(DOCX)

S3 Table Time requirement for inspection and sampling for taxa inventory in 6 forest target areas using different sampling methods. Time requirement calculated in hours/km^2^ area for 3 seasonal inspection rounds. Target area sizes correspond to the compartments CA2acorr (fully accessible area without forest edge hem). O-map method: along all ways and between all ways (complete); NT-map method: only along NT-ways; all taxa were recorded separately for the compartments of the three sampling methods. Further information on the inspection and sampling is given under “Material and methods”.
(DOCX)

S4 Table Comparison of the various elements on O-maps and NT-maps relevant for plant inventories for the 6 forest target areas. **Table A in S4 Table:** Comparison of area sizes and numbers of subareas on O-maps and NT-maps. CA2 = accessible; TA = total). Subareas are bounded by ways, other lines visible in forest and on O-maps (such as paths, traces, fences, vegetation boundaries, water courses, earth walls, gullies, etc.) and target area boundaries; Subareas between ways on NT-map are bounded by ways and target area boundaries. **Table B in S4 Table.** Comparison of way lengths on O-maps and NT-map. **Table C in S4 Table:** Comparison of numbers and frequencies of small objects (features) on O-maps and NT-map.
(DOCX)

S5 Table Relative rareness of plant taxa only found “between all ways” for all 6 target areas. “between all ways” means, that these taxa were only detected by the O-map method. Measure of relative rareness “Taxa distribution <40%” counts relatively rare taxa present in less than 40% of the total of 593 subareas completely covering the area of Switzerland [14]. Measure of relative rareness “Abundance class 1–25” is based on local rareness with respect to the target area; taxa with 1–25 individual plants per target area are counted as relatively rare.
(DOCX)

S6 Table Reproducibility in the detection of plant sites by the O-map method in two inspection rounds. Numbers of sites for 5 plant species were recorded by one investigator during 2 consecutive inspection rounds in summer of one year in forest target area 714f (30.1 ha). 1^st^ inspection round = A; 2^nd^ inspection round = B. Further information on inspection and sampling is given under “Material and methods”.
(DOCX)

S7 Table Presence of the found plant taxa in the compartments of the 6 forest target areas. Plant taxa designation according to Lauber et al. [14]. Abundance per target area in classes W, I (1–25 plants), W, II (26–1000 plants), W, III (>1000 plants). Presence of taxa: 1 = taxon present;— = taxon not present. Compartment designation: B = along NT-ways; R = along O-ways; G = between ways.
(XLSX)

S8 Table Presence of the relatively rare plant taxa found for “only between all ways”. Plant taxa designation according to Lauber et al. [14]. Criteria for relative rareness: "Ab 1–25" = Abundance is 1–25 plants in the corresponding target area; "< 40%" = present in less than 40% of 593 subareas of Switzerland, described by Lauber et al. [14]. Presence of the rel. rare plant taxa "only between all ways" with "Ab 1–25" is indicated by yellow cells, those with "<40%" by dark yellow cells.
(XLSX)
