# Supplementary material for: A method for complete plant taxon and site inventories in large forest areas with the help of orienteering maps, as exemplified by target forests in Switzerland
Source: PLoS One. 2019 Dec 10;14(12):e0225927. doi: 10.1371/journal.pone.0225927 (PMC6903739; doi:10.1371/journal.pone.0225927)
Supplement: S1 Table — O-maps edited by OLG Bern: www.olgbern.ch; NT-map edited by swisstopo: www.swisstopo.admin.ch. (DOCX) [file pone.0225927.s001.docx]

|  | | | | |
| --- | --- | --- | --- | --- |
| Target area | Coordinates (Swiss Grid) | Inventory area (CA2corr) | O-map  (orienteering map) | NT-map  (national topographic map) |
| Designation | X-axis  Y-axis | Size (ha) | Map name, map number,  editor, year, scale | Map name, map number,  editor, year, scale |
| 703f | 590000  198000 | 71.46 | “Forst”, #1508 Q,  OLG Bern, (2011), 1:10 000 | Landeskarte der Schweiz, “Bern”, #1166,  swisstopo, (2012), 1:25 000 |
| 809f | 596000  197000 | 70.29 | “Könizberg”, #299 Q,  OLG Bern, (2013), 1:10 000 | Ditto (as above) |
| 410f | 597000  201000 | 88.42 | “Bremgartenwald”, #492 Q,  OLG Bern, (2009), 1:10 000 | Ditto (as above) |
| 710f | 597000  198000 | 80.59 | “Könizberg”, #299 Q,  OLG Bern, (2013), 1:10 000 | Ditto (as above) |
| 810f | 597000  197000 | 66.99 | “Könizberg”, #299 Q,  OLG Bern, (2013), 1:10000 | Ditto (as above) |
| 714f | 601000  198000 | 30.19 | “Dählhölzli”, #1772 Q,  OLG Bern, (2014), 1:5 000 | Ditto (as above) |
