# Supplementary material for: A method for complete plant taxon and site inventories in large forest areas with the help of orienteering maps, as exemplified by target forests in Switzerland
Source: PLoS One. 2019 Dec 10;14(12):e0225927. doi: 10.1371/journal.pone.0225927 (PMC6903739; doi:10.1371/journal.pone.0225927)
Supplement: S2 Table — Further specification of the compartments is given under “Material and methods”. (DOCX) [file pone.0225927.s002.docx]

|  | | | | | | | |
| --- | --- | --- | --- | --- | --- | --- | --- |
|  |  | Area size (ha) | | | | | |
| Compartment | Accessibiliy | 703f | 809f | 410f | 710f | 810f | 714f |
| TA | Total forest area | 74.31 | 70.67 | 92.84 | 84.68 | 67.31 | 36.55 |
| CA1 | Not accessible | 2.51 | 0 | 4.32 | 3.82 | 0 | 5.99 |
| CA2 | Accessible | 71.80 | 70.67 | 88.52 | 80.86 | 67.31 | 30.56 |
| CA2a | Fully accessible | 57.75 | 55.54 | 69.35 | 57.99 | 45.99 | 30.56 |
| CA2b | Reduced accessible | 14.05 | 15.13 | 19.17 | 22.86 | 21.31 | 0 |
| CA2corr | Accessible corrected | 71.46 | 70.29 | 88.42 | 80.59 | 66.99 | 30.19 |
| CA2acorr | Fully accessible corrected | 57.41 | 55.16 | 69.25 | 57.73 | 45.67 | 30.19 |
