# Supplementary material for: A method for complete plant taxon and site inventories in large forest areas with the help of orienteering maps, as exemplified by target forests in Switzerland
Source: PLoS One. 2019 Dec 10;14(12):e0225927. doi: 10.1371/journal.pone.0225927 (PMC6903739; doi:10.1371/journal.pone.0225927)
Supplement: S3 Table — Time requirement calculated in hours/km2 area for 3 seasonal inspection rounds. Target area sizes correspond to the compartments CA2acorr (fully accessible area without forest edge hem). O-map method: along all ways and between all ways (complete); NT-map method: only along NT-ways; all taxa were recorded separately for the compartments of the three sampling methods. Further information on the inspection and sampling is given under “Material and methods”. (DOCX) [file pone.0225927.s003.docx]

|  | | | | | | | |
| --- | --- | --- | --- | --- | --- | --- | --- |
| Sampling method | Inspection + sampling time per fully accessible area [hours/km²] (in % of values by O-map method) | | | | | | |
| Target area-size in [ha] | 703f-57.4 | 809f-55.2 | 410f-69.3 | 710f-57.7 | 810f-45.7 | 714f-30.2 | Mean±SD |
| O-map method | 260.8 (100) | 248.5 (100) | 241.9 (100) | 269.4 (100) | 208.7 (100) | 238.3 (100) | 244.6±21.1 (100) |
| NT-map method | 082.6 (31.7) | 109.7 (44.2) | 111.4 (46.0) | 094.9 (35.2) | 072.1 (34.6) | 065.6 (27.5) | 089.4±19.2 (36.5) |
| At NT- and O-ways only | 133.4 (51.2) | 148.5 (59.8) | 145.2 (60.0) | 136.9 (50.8) | 112.8 (54.0) | 114.5 (48.1) | 131.9±15.2 (54.0) |
|  |  |  |  |  |  |  |  |
|  |  |  |  |  |  |  |  |
