# Supplementary material for: A method for complete plant taxon and site inventories in large forest areas with the help of orienteering maps, as exemplified by target forests in Switzerland
Source: PLoS One. 2019 Dec 10;14(12):e0225927. doi: 10.1371/journal.pone.0225927 (PMC6903739; doi:10.1371/journal.pone.0225927)
Supplement: S4 Table — Table A in S4 Table: Comparison of area sizes and numbers of subareas on O-maps and NT-maps. CA2 = accessible; TA = total). Subareas are bounded by ways, other lines visible in forest and on O-maps (such as paths, traces, fences, vegetation boundaries, water courses, earth walls, gullies, etc.) and target area boundaries; Subareas between ways on NT-map are bounded by ways and target area boundaries. Table B in S4 Table. Comparison of way lengths on O-maps and NT-map. Table C in S4 Table: Comparison of numbers and frequencies of small objects (features) on O-maps and NT-map. (DOCX) [file pone.0225927.s004.docx]

|  | Subarea size in ha (mean±SD)  Number of subareas (total/> 1 ha) | | | | | | | | | | | | | |  |
| --- | --- | --- | --- | --- | --- | --- | --- | --- | --- | --- | --- | --- | --- | --- | --- |
| Target area | 703f | | 809f | | 410f | | 710f | | 810f | | 714f | | Mean±SD | | |
| Between lines (O-map; CA2) | 0.18±0.19  319/2 | | 0.10±0.10  582/0 | | 0.24±0.23  290/6 | | 0.20±0.18  292/2 | | 0.14±0.16  320/1 | | 0.25±0.22  121/2 | | 0.19±0.06 | | |
| Between ways (O-map; TA) | 0.96±1.54  75/19 | | 1.09±1.40  65/23 | | 1.05±1.60  84/26 | | 0.89±1.21  91/27 | | 0.83±1.10  81/24 | | 0.31±0.28  98/4 | | 0.85±0.28 | | |
| Between ways (NT-map; TA) | 2.05±2.30  35/19 | | 1.72±1.81  41/23 | | 2.01±2.44  44/21 | | 1.93±1.80  42/24 | | 1.73±1.32  39/25 | | 1.39±1.08  22/13 | | 1.80±0.25 | | |
|  | | | | | | | | | | | | | | |  |
|  | | Total way lengths in km [km]  Total way lengths in km per target area (CA2) in km² [km/ km²] | | | | | | | | | | | | |  |
| Target area | | 703f | 809f | | 410f | | 710f | | 810f | | 714f | | Mean±SD | |  |
| On O-maps: Total way length  Way length/area | | 8.34  11.62 | 12.80  18.11 | | 14.24  16.09 | | 16.18  20.01 | | 12.31  18.28 | | 10.68  34.93 | | 19.84±7.93 | |  |
|  | |  |  | |  | |  | |  | |  | |  | |  |
| On NT-map: Total way length  Way length/area | | 5.00  6.96 | 9.46  13.39 | | 10.19  11.51 | | 9.45  11.68 | | 7.52  11.17 | | 4.50  14.72 | | 11.57±2.63 | |  |

|  | Number of small objects [n]  Number of small objects (n) per target area (CA2) in km²) [n/km²] | | | | | | |
| --- | --- | --- | --- | --- | --- | --- | --- |
| Target area | 703f | 809f | 410f | 710f | 810f | 714f | Mean±SD |
| Objects on O-maps:  Number (n)  Number/area | 99 137.9 | 175 247.6 | 98  110.7 | 139  173.2 | 79  117.4 | 139  454.8 | 206.9±131.4 |
|  |  |  |  |  |  |  |  |
| Objects on NT-map:  Number (n)  Number/area | 1  1.3 | 1  1.4 | 1  1.1 | 0  0 | 0  0 | 0  0 | 0.7±0.7 |
