# Supplementary material for: A method for complete plant taxon and site inventories in large forest areas with the help of orienteering maps, as exemplified by target forests in Switzerland
Source: PLoS One. 2019 Dec 10;14(12):e0225927. doi: 10.1371/journal.pone.0225927 (PMC6903739; doi:10.1371/journal.pone.0225927)
Supplement: S5 Table — “between all ways” means, that these taxa were only detected by the O-map method. Measure of relative rareness “Taxa distribution <40%” counts relatively rare taxa present in less than 40% of the total of 593 subareas completely covering the area of Switzerland [14]. Measure of relative rareness “Abundance class 1–25” is based on local rareness with respect to the target area; taxa with 1–25 individual plants per target area are counted as relatively rare. (DOCX) [file pone.0225927.s005.docx]

|  | | | | | | | | | |
| --- | --- | --- | --- | --- | --- | --- | --- | --- | --- |
| Measure of relative rareness | Percentage of relatively rare taxa among all new taxa found “between all ways” | | | | | | | | |
| Target area | | | 703f | 809f | 410f | 710f | 810f | 714f | Mean±SD |
| Taxa distribution <40% | | 26.3 | | 45.9 | 29.4 | 42.0 | 31.4 | 27.7 | 33.8±8.2 |
| Abundance class 1-25 | | 68.9 | | 78.6 | 78.0 | 68.3 | 86.4 | 68.9 | 74.9±7.4 |
|  | |  | |  |  |  |  |  |  |
