# Supplementary material for: A method for complete plant taxon and site inventories in large forest areas with the help of orienteering maps, as exemplified by target forests in Switzerland
Source: PLoS One. 2019 Dec 10;14(12):e0225927. doi: 10.1371/journal.pone.0225927 (PMC6903739; doi:10.1371/journal.pone.0225927)
Supplement: S6 Table — Numbers of sites for 5 plant species were recorded by one investigator during 2 consecutive inspection rounds in summer of one year in forest target area 714f (30.1 ha). 1st inspection round = A; 2nd inspection round = B. Further information on inspection and sampling is given under “Material and methods”. (DOCX) [file pone.0225927.s006.docx]

|  | | | | | |
| --- | --- | --- | --- | --- | --- |
| Inspection round | Number of sites found (in % of the total of sites) | | | | |
| Species | *Prunus laurocerasus* | *Sorbus aucuparia* | *Pinus sylvestris* | *Athyrium filix-femina* | *Dryopteris filix-mas* |
| Rounds A and B | 106 (69.7) | 168 (66.1) | 187 (94.0) | 60 (61.2) | 110 (72.8) |
| Round A only | 14 (09.2) | 23 (09.1) | 4 (02.0) | 15 (15.3) | 17 (11.3) |
| Round B only | 32 (21.1) | 63 (24.8) | 8 (04.0) | 23 (23.5) | 24 (15.9) |
| Round A or B (Total) | 152 (100.0) | 254 (100.0) | 199 (100.0) | 98 (100.0) | 151 (100.0) |
